# Morphological observations on the role of HERS cells in cementum development of rabbit molars

**DOI:** 10.3389/fbioe.2025.1659704

**Published:** 2025-09-24

**Authors:** Xinping Zhan, Licheng Xing, Lin Meng, Yingying Cheng, Junjun Wang, Qiuxu Wang, Xiaolin Sun

**Affiliations:** ^1^ School of Stomatology, Dalian University, Dalian, China; ^2^ Department of Stomatology, Zhongshan Affiliated Hospital of Dalian University, Dalian, China

**Keywords:** cementum, epithelial stem cells, epithelial-mesenchymal transition, periodontal ligament, rabbit molars

## Abstract

**Objective:**

To investigate the relationship between cementum development and epithelial stem cells in rabbit molars, as well as the associated morphological characteristics.

**Methods:**

A total of 25 New Zealand white rabbits were selected and divided into five age groups (14 days, 21 days, 28 days, 50 days, and 8 months). The developmental process of cementum was examined using micro-computed tomography (micro-CT), scanning electron microscopy (SEM), and histological methods.

**Results:**

Cementum in rabbit molars was classified into three types: acellular extrinsic fiber cementum (AEFC), cellular intrinsic fiber cementum (CIFC), and cellular mixed stratified cementum (CMSC). With increasing age, both the bone volume fraction (BV/TV) and bone mineral density of the cementum significantly increased (P < 0.05). Following apoptosis of epithelial cells, mesenchymal stem cells (MSCs) differentiated into cementoblasts; however, the role of epithelial-mesenchymal transition (EMT) was found to be limited. The formation of functional cementum primarily depended on the insertion of periodontal ligament (PDL) fibers.

**Conclusion:**

The cementum of rabbit molars surrounds the enamel. With increasing age, both the volume and density of the cementum continue to increase, and its functions differ significantly based on its classification. EMT is not the principal mechanism involved.

## 1 Introduction

Cementum is a hard connective tissue that covers the surface of the tooth root and plays a vital role in tooth attachment and periodontal function (Yamamoto et al., 2016). From the 17th to the 19th century, cementum was often conflated with bone tissue. It was not until the 1830s, with the rapid advancement of microscopy and histological techniques, that Jan Purkinje and Anders Retzius provided the first detailed description of human cementum. They defined two distinct types of cementum: acellular and cellular, and identified the presence of intrinsic cementoblasts embedded within cellular cementum. Subsequently, Jones (Yamamoto et al., 2016) emphasized the functional significance of fiber insertion into cementum. As a result, the two fiber types associated with cementum—extrinsic (Sharpey’s) fibers embedded as principal fibers, and intrinsic fibers that form part of the cementum matrix—were incorporated into the classification system of cementum. Schroeder (Gibbins et al., 2002) later proposed a classification that includes acellular extrinsic fiber cementum (AEFC), cellular intrinsic fiber cementum (CIFC), cellular mixed stratified cementum (CMSC), acellular afibrillar cementum (AAC), and acellular intrinsic fiber cementum (AIFC). This classification system has since been widely adopted in the field of dentistry.

Although significant advances have been made in understanding cementum development and regeneration at the molecular biology level, its cellular origins and developmental mechanisms remain unclear. Cementum regeneration continues to be a major unresolved challenge in the field of dental tissue engineering (Raja et al., 2009; Vaquette et al., 2018; Duan et al., 2020; Shalehin et al., 2022).

In the field of tooth regeneration research, teeth of rodents with continuous growth capacity have garnered significant attention from researchers. The incisors of mice and rats harbor persistently viable ameloblasts, mesenchymal cells, and the Hertwig’s epithelial root sheath (HERS)—a structure recognized as a pivotal regulatory core for tooth development and sustained renewal (Bi et al., 2023). As an odontogenic epithelial tissue, HERS not only participates in the signaling regulation of root morphogenesis and dentin formation, but its crosstalk with mesenchymal cells has also been identified as a key molecular mechanism underlying the continuous growth of rodent incisors (Thesleff and Tummers, 2009; Hazrati et al., 2024). Of particular importance is that, during conventional root development, HERS induces the differentiation of adjacent dental follicle mesenchymal cells into cementoblasts by secreting signaling molecules such as BMP and TGF-β, thereby initiating the formation and mineralization of cementum (Bosshardt et al., 2015; Luder, 2015). To date, numerous studies have employed rodent incisors as models to investigate the mechanisms governing tooth growth (Li et al., 2017; Fresia et al., 2021). However, given the extremely low cementum content in rodent incisors, coupled with the incomplete understanding of the existence form and functional properties of HERS in incisors—for instance, whether it retains the classical functional mode of inducing cementum formation or engages in the limited cementum production of incisors via a specialized mechanism—research specifically focusing on the cementum regeneration potential in the continuously growing incisors of mice or rats remains relatively scarce.

With the continued advancement of dental histology, increasing attention has been directed toward the regeneration of cementum. As a component of the periodontal complex (cementum–periodontal ligament–alveolar bone), cementum is an indispensable structure in all thecodont animals. However, substantial interspecies differences exist among mammals in terms of the distribution, cellular architecture, and attachment mode of cementum (Sun et al., 2024). In well-known brachyodont dentitions, the length of the tooth root exceeds that of the crown. The root is covered with cementum, which anchors the tooth to the alveolar bone via the periodontal ligament (PDL) (Boyde, 2005). However, not all animals exhibit cementum restricted to the root surface. In many herbivorous animals with hypsodont dentitions—such as those in the genera *Equus* and *Elephas*—the crown continues to grow even after the root is formed, resulting in a crown length that exceeds the root length. Part of the enamel-covered crown is embedded in the alveolar bone. In such cases, cementum covers not only the radicular dentin but also the coronal enamel, thereby connecting the tooth to the alveolar bone via the PDL (Damuth and Janis, 2011; Sahara, 2014; Englisch et al., 2017; Pöschke et al., 2018). In certain rodent species, such as rabbits and guinea pigs, the molars exhibit continuous growth throughout the animal’s lifespan. Similar to the incisors of rats, their enamel organs remain deeply embedded within the jawbone and do not undergo regression or degradation. Consequently, these animals possess teeth with enamel-covered crowns but lack true anatomical roots. Such dentition is classified as hypselodont (Müller et al., 2015; Gomes Rodrigues et al., 2017). What has drawn particular interest is that, despite the absence of roots, hypselodont molars still exhibit substantial amounts of cementum covering the outer surface of the enamel. This cementum plays a crucial role in anchoring the tooth to the alveolar bone.

Would the function of cementum in the molars of hypselodont animals be consistent with that in brachydont teeth such as those of humans? Does it continue to grow in conjunction with tooth eruption? Is cementum development influenced by epithelial stem cells or mesenchymal stem cells, and could these persistently existing stem cells serve as a cellular source for cementogenesis (Itaya et al., 2017; Yang and Cho, 2020; Yang et al., 2020; Gibbins et al., 2002)? To address these questions, we sought to investigate the developmental dynamics of cementum in the molars of hypselodont animals. Therefore, this study employed rabbit molars—teeth characterized by continuous growth—as a model to conduct morphological observations on cementum development. The aim was to elucidate the relationship between persistently existing stem cell niches and cementogenesis, thereby providing insights that may contribute to advances in the field of human cementum regeneration engineering.

## 2 Materials and methods

### 2.1 Experimental animals and grouping

A total of 25 New Zealand white rabbits used in this study were all offspring of breeding rabbits housed at the Laboratory Animal Center of the Affiliated Zhongshan Hospital of Dalian University, Dalian, China (they were not entirely from the same litter to reduce litter effects). All experimental procedures were approved by the Animal Experimentation Center and the Institutional Animal Care and Use Committee (IACUC) of this hospital, and complied with the Guidelines for Ethical Review of Animal Experiments (GB/T 35892-2018).

Newborn rabbits born concurrently were implanted with medical plastic ear tags within 7 days after birth. During the ear tag implantation, group allocation was completed based on the unique serial number on each ear tag and a random number table generated by SPSS 17.0. After matching the ear tag numbers with the random sequence, the rabbits were sorted in ascending order of the random sequence and evenly assigned to 5 groups (5 rabbits per group). This ensured that group allocation and individual identification were completed simultaneously, reducing the interference of environmental factors on inter-group consistency during subsequent rearing. Before weaning (from birth to 28 days of age), the rabbits in each group were still housed together with their mothers in dedicated mother-offspring cages—this co-housing period allowed the young rabbits to obtain nutritional support from maternal milk, ensuring normal early development and conforming to the conventional rearing standards for experimental New Zealand white rabbits. After weaning at 28 days of age, the young rabbits in each group were separated from their mothers and housed individually or in groups in cages to avoid maternal interference and ensure the young rabbits could adapt to solid feed independently. All rabbits were reared in a standardized breeding room meeting the standards of Laboratory Animal Environment and Facilities (GB 14925-2010), with the rearing environment controlled at a temperature of 20 °C–26 °C, relative humidity of 40%–70%, and a light cycle of 12 h of light/12 h of darkness. Bedding was changed regularly, and sterile feed and drinking water were provided (sterile solid feed was given after weaning to meet nutritional needs).

### 2.2 Specimen preparation

Rabbits in the 5 groups were euthanized by intravenous injection of an excessive dose of sodium pentobarbital via the ear marginal vein at 14 days, 21 days, 28 days, 50 days, and 8 months of age, respectively. The dosage was 100–150 mg/kg body weight (120–150 mg/kg for 14-day-old young rabbits and 100–120 mg/kg for adult rabbits). After confirming the cessation of respiration and heartbeat, target tissues/organs were collected in accordance with aseptic operation standards.

The mandible was carefully dissected and separated, and bone blocks containing the first mandibular molar along with the surrounding alveolar bone were excised. The specimens were immediately fixed in 4% paraformaldehyde (prepared in 0.1 M phosphate buffer, pH 7.4) at 4 °C overnight.

### 2.3 Micro-CT analysis

Rabbit mandible Micro-CT analysis was performed using a Scanco μCT35 (Scanco Medical, Bassersdorf, Switzerland). Rabbit molar specimens were mounted on a carbon fiber plate and positioned at the center of the scanning field. Micro-CT scanning was performed using the Inveon Acquisition Workplace system with the following parameters: tube voltage 80 kV, tube current 500 μA, exposure time 1,600 m, frame averaging number 1, pixel binning factor 1, effective pixel size 9.41 μm, rotation angle 360°, and 360 projection steps. Data reconstruction was conducted using the Inveon Acquisition Workplace software.

### 2.4 Scanning electron microscopy (SEM) observation

Mandibular first molar specimens from the 50-day and 8-month groups were fixed with freshly prepared 4% paraformaldehyde in PBS (pH 7.4). After graded ethanol dehydration, specimens were embedded in methyl methacrylate (MMA) resin. The embedded blocks were sectioned along the horizontal plane of the mandible using a water-cooled diamond circular saw. The specimen surfaces were sequentially polished with 1 μm, 0.3 μm, and 0.05 μm alumina α-polishing suspensions using soft cloth polishing wheels. After gold-palladium sputter coating, the specimens were examined and analyzed using scanning electron microscopy (JEOL Limited, Tokyo, Japan).

### 2.5 Histological analysis

After decalcification with 10% ethylenediaminetetraacetic acid (EDTA), the specimens were dehydrated in a graded ethanol series and embedded in paraffin. Serial transverse sections of the mandibular first molars were cut at a thickness of 5 μm. Some sections were stained with hematoxylin and eosin (H&E) for routine histological observation, while the remaining sections were used for immunohistochemistry (IHC), TUNEL assay, and PCNA detection. All histomorphometric analyses were performed with Bioquant Osteo software (Bioquant Image Analysis, Nashville, TN, United States).

The concentrations of the primary antibodies for the immunohistochemistry were rabbit polyclonal anti-PCNA(1:200, GB11010-100,Wuhan Servicebio Technology Co., Ltd.), CF488 Tunel Cell Apoptosis Detection Kit (1:200,G1504-50T,Wuhan Servicebio Technology Co., Ltd.), Anti-Cytokeratin 14 Rabbit pAb (K14, 1:500; GB11803-100,Wuhan Servicebio Technology Co., Ltd.), Anti-Alkaline Phosphatase Rabbit pAb (TNALP,1:400; GB11527-100,Wuhan Servicebio Technology Co., Ltd.),Anti-Vimentin Rabbit pAb (Vimentin,1:400; GB11192-100,Wuhan Servicebio Technology Co., Ltd.).

### 2.6 Statistical analysis

The experimental results were expressed as “mean ± standard deviation” (mean ± SD). The significance of differences was determined using SPSS 17.0 software (SPSS, Inc., United States) via one-way analysis of variance (1-way ANOVA) combined with Tukey’s HSD *post hoc* tests. A P-value <0.05 was considered statistically significant.

## 3 Results

### 3.1 Molar morphology

**FIGURE 1:**
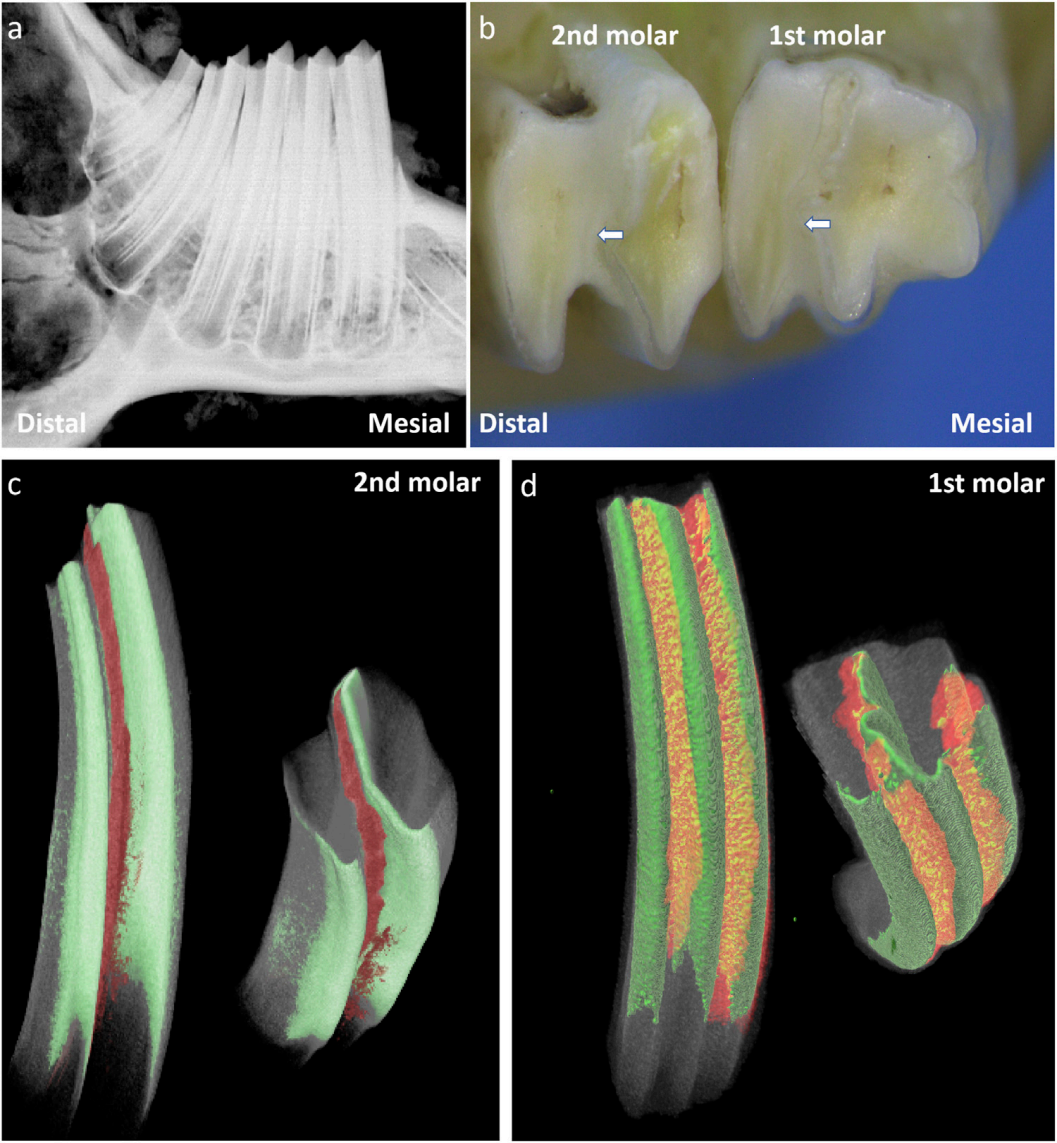
A schematic images of the structure of rabbit molars for 50 days. Note: **(a)** Radiograph of the rabbit mandibular molars; **(b)** Occlusal view of the mandibular first molar and second M, the white arrows indicate the enamel folding areas formed by enamel invagination in the rabbit’s molar.; **(c)** 3D Reconstructed Image of the Second Mandibular Molar of Rabbit after 3D Segmentation (Masking) of CT Data; **(d)** 3D Reconstructed Image of the First Mandibular Molar of Rabbit after 3D Segmentation (Masking) of CT Data; . The green area indicates enamel, The red area represents the cellular cementum that has invaginated into the enamel folding area.

Rabbit molars are classified as hypselodont teeth and harbor epithelial stem cell niches beneath the enamel crown. Radiographic imaging showed that rabbit molars lack a true root apex. The first mandibular molar presents with three cusps on the buccal side, whereas the other mandible molars exhibit two cusps. The enamel folds inward from the buccal side to form hard ridges, dividing the tooth into mesial and distal cusps (Figure 1). The dentin and enamel of these two parts are nearly completely separated and connected only by a small amount of enamel on the mesiolingual side. Micro-CT analysis revealed that cementum extends into the enamel fold regions, linking the otherwise separated cusps.

### 3.2 Distribution characteristics of cementum

**FIGURE 2:**
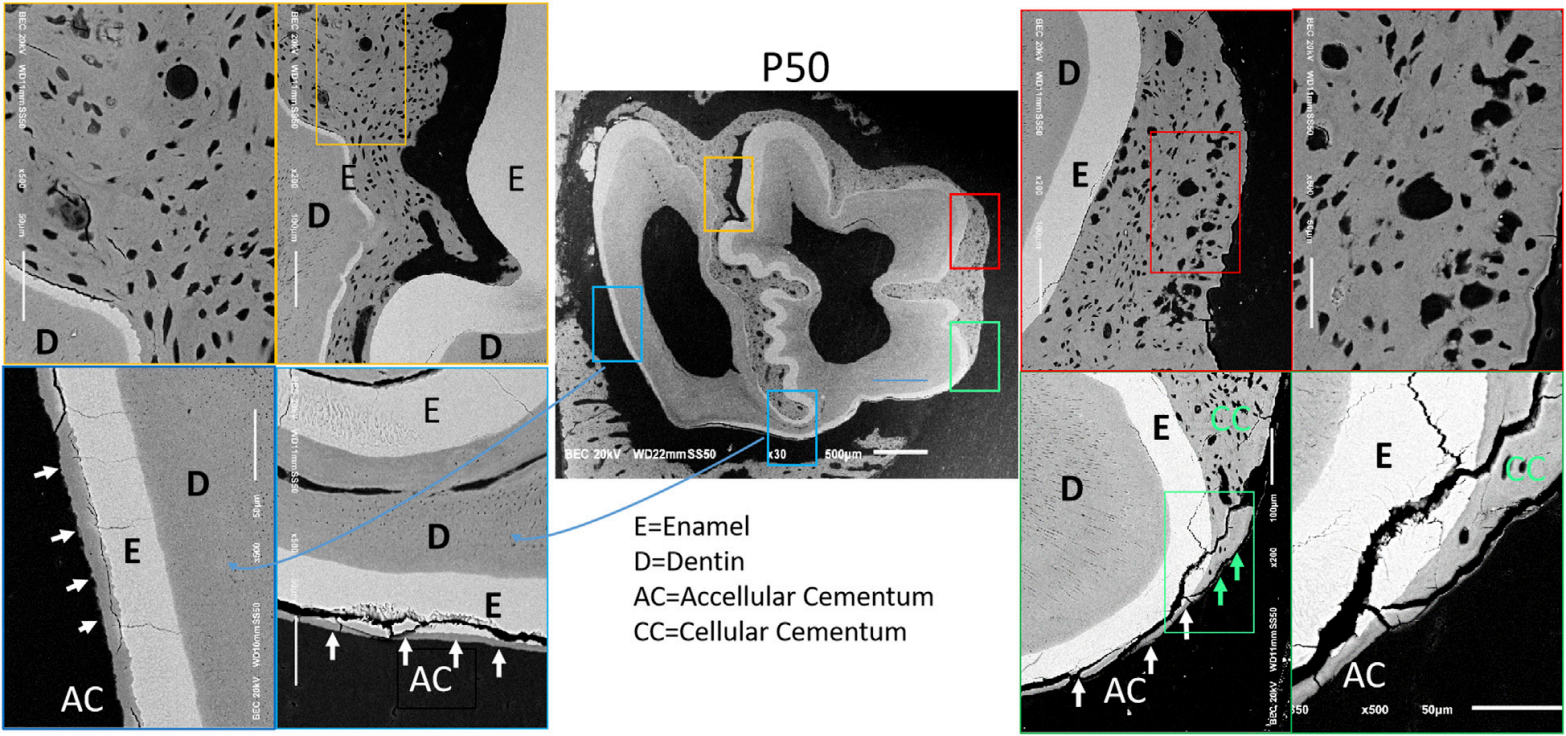
Distribution images of cementum of 50-day rabbit first molar. Note: 1) D = Dentin; 2) E = Enamel; 3) AC = Acellular Cementum; 4) CC = Cellular Cementum.

Scanning electron microscopy (SEM) observation of the four regions in the first mandibular molar of 50-day-old rabbits showed the following: cellular component-rich cementum was present in the enamel folding area (yellow border); numerous cells and cementoid structures were observed on the mesial side (red border); acellular cementum was found on the lingual and distal enamel surfaces (blue border); and the transition from cellular cementum to acellular cementum could be seen at the mesiolingual axial angle (green border) (arrows) (Figure 2). These findings indicate an asymmetric distribution of cementum in the first mandibular molar of rabbits: cellular cementum is mainly located on the labial and mesial surfaces, while acellular cementum dominates the lingual and distal surfaces.

### 3.3 Micro-CT observations

**FIGURE 3:**
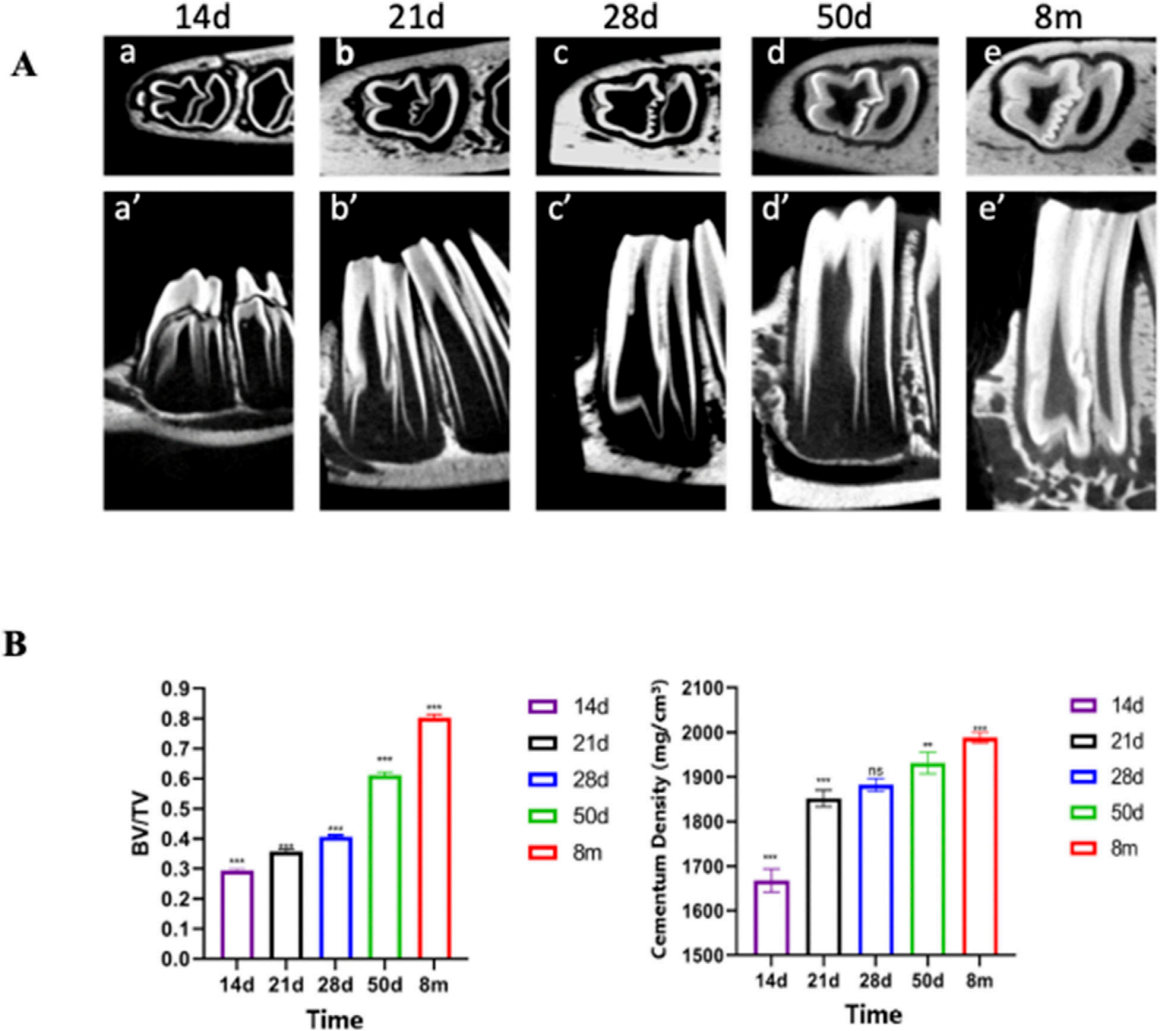
Micro-CT Reveals Significant Thickening and Density Increase in Rabbit Cementum. **(A)** CT scan images of rabbit teeth and Bone volume fraction Note: a–e represent transverse sections; a′–e′ represent sagittal sections. **(B)** Data are presented as mean ± standard deviation (n = 5). One - way ANOVA was performed to assess overall group differences, followed by Tukey’s HSD post - hoc tests for pairwise comparisons. Note: a:vs. P14 P < 0.001; b:vs. P28 P < 0.01; c:vs. P28 P < 0.001; d:vs. P50 P < 0.05.

The CT scans of the bone blocks at 14 days, 21 days, 28 days, 50 days and 8 months were completed, and the stem cell pools could be seen on the root side of the rabbit’s teeth and the dentin gradually became thicker and more abundant with the increase of time, and the dentin was more obvious at 50 days and 8 months, and the deciduous teeth were not yet detached at 14 days, and the dentin in the same area was analyzed by CT (Figure 3A). At P28, there was a small mass of cellular cementum lining the first molar root surface.

Starting from P28, there was a gradual and steady increase in the mass and density of cellular cementum. Micro-CT quantitative analyses (Figure 3B; n = 5) further confirmed these temporal changes: indices measured at P50 and 8M exhibited significant differences compared to earlier developmental periods (P14, P21, P28). Specifically, 8M and P50 values differed significantly from P14 (denoted as “a” for P < 0.001), with 8M additionally showing significant differences from P28 (denoted as “c” for P < 0.001 for 8M vs. P28) and P50 (denoted as “d” for P < 0.05 for 8M vs. P50). P50 also differed significantly from P28 at the P < 0.01 level (denoted as “b”). These statistical differences validate the progressive thickening and maturation of dentin and cellular cementum observed qualitatively.

### 3.4 Scanning electron microscopy analysis

**FIGURE 4:**
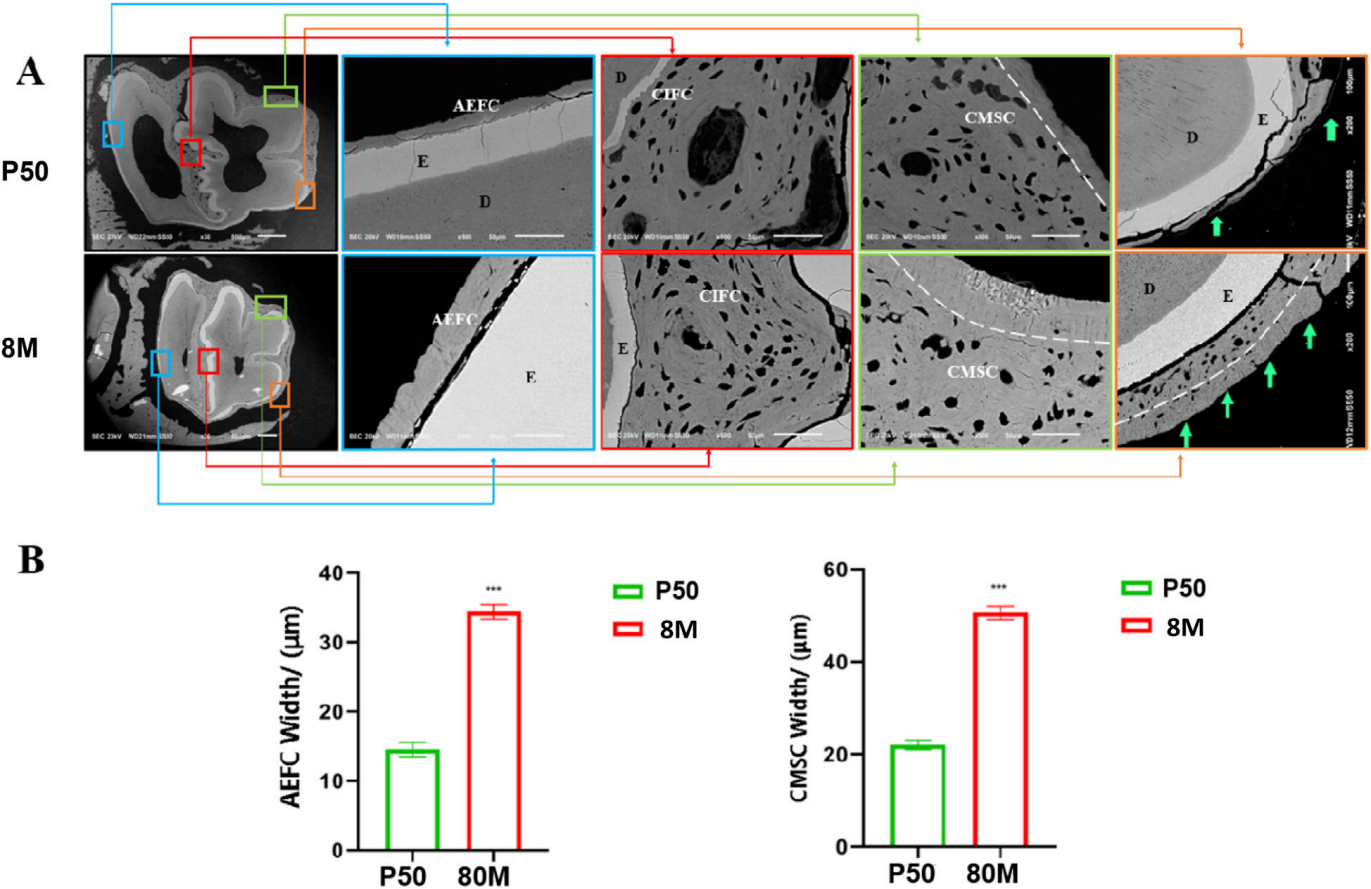
Scanning electron microscope images of rabbit mandibular first molars at 50 days and 8 months. **(A)** Note: 1) AEFC = acellular extraneous fibrous cementum; 2) CIFC = cellular intrinsic fibrous cementum;3)CMSC= cellular mixed stratified cementum; 4) E= enamel; 5) D = dentin. **(B)** Note: *** indicates P < 0.001 with statistical significance; the comparison of cementum width between the two groups showed a significant difference (P < 0.001).

Based on the CT data, we selected the mandibular first molars of rabbits at P50 and 8 months of age, where the most significant changes were observed, for cross-sectional scanning electron microscopic analysis. Within the full cross-sectional images, four regions were chosen from left to right for magnified observation and were labeled with distinct colors—specifically blue, red, green, and orange in left-to-right order. These labeled regions are presented sequentially on the right side of the cross-sectional images. It was observed that the morphology and structure of cementum in rabbit molars varied across different regions. Herein, the cementum of rabbit molars was labeled in accordance with the classification system proposed by Yamamoto (Yamamoto et al., 2016) (Figure 4A). (1) The blue region corresponds to the acellular cementum located on the outer side of the enamel on the distal surface of the distal cusp of the rabbit mandibular first molar. This portion of cementum is devoid of cells and is connected to the periodontal ligament; thus, we consider it to be part of the acellular extrinsic fiber cementum (AEFC). Moreover, with increasing age, the width of AEFC increases significantly (Figure 4B); (2) The red region corresponds to the cellular intrinsic fiber cementum (CIFC) located between the enamel of the mesial and distal cusps of the rabbit molar. Since it is sandwiched between the enamel on both the left and right sides and does not come into contact with the periodontal ligament at all, we consider that no exogenous periodontal ligament fibers will penetrate into it; (3) The green region corresponds to the cellular cementum located at the enamel fold of the mesial buccal cusp. Its outermost layer is connected to the periodontal ligament. As observed, it contains abundant cells and has exogenous periodontal ligament fibers penetrating into it; thus, we consider it to be part of the cellular mixed stratified cementum (CMSC). And obviously, the surface of CMSC exhibits a lamellar acellular cementum structure. Furthermore, with increasing age, this lamellar structure thickens significantly (p < 0.001) (Figure 4B). (4) The orange region is located at the angle where the mesial surface and lingual surface of the mesial cusp meet. The cementum on the lingual surface was distinctly acellular at 50 days, but transformed into mixed cementum by 8 months of age. Moreover, the outermost layer, which connects to the periodontal ligament, still exhibits a lamellar structure.

### 3.5 Histological observations

**FIGURE 5:**
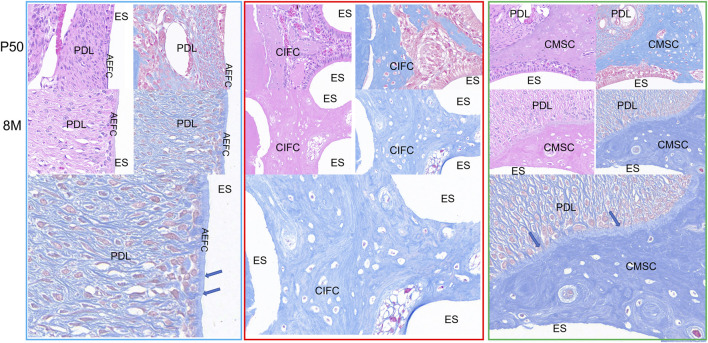
Cementum distribution images of mandibular first molar in rabbits at 50 days and 8 months. Note: AM: Ameloblasts; AEFC: Acellular extrinsic fiber cementum; AB: Alveolar bone; CIFC: Cellular intrinsic fiber cementum; CMSC: Cellular mixed stratified cementum; D: Dentin; ES: Enamel space; PDL: Periodontal ligament; The blue arrows represent Sharpey’s fibers.

In order to clearly understand the relationship between rabbit dentin and epithelial cells, HE and Masson staining analysis was performed on the mandibular first molar of rabbits at 50 days and 8 months, we chose these three positions to observe, the blue area HE staining can be observed in the cell-free exogenous fibrous odontoblasts (Acelluar extrinsic fibre cementum, AEFC), the outer side of the enamel-forming cells that are arranged, at 8 months, the Enamel-forming cells gradually differentiated into AEFC, red area HE staining can see obvious cells within it, and blood vessels can be seen within it, i.e., cellular intrinsic fibrous cementum (CIFC), and at 8 months, the mineralization of CIFC was more obvious, and enamel-forming cells disappeared, and cellular mixed stratified cementum (CMSC) can be seen by HE staining in the green area, and the periodontal fibers can be seen to be evenly distributed in the CMSC. The enamel cells disappeared at 8 months (Figure 5). The AEFC in the blue area were seen to be penetrated by periodontal fibers in the Masson’s trichrome stain, however, there were no periodontal fibers in the red area in the Masson’s trichrome stain, and their distribution was not homogeneous, and in the green area, periodontal fibers were seen to be penetrated into the CMSC in the Masson’s trichrome stain, which was even more obvious at 8 months, and the increase in the width of the CMSC was concentrated in the area where periodontal fibers had been penetrated, and the mineralization of the outer side of the CMSC was also visible (Blue arrow). The mineralization of the outer side of the CMSC increased significantly and the width widened significantly, while the side close to the enamel cells did not show a significant change in width and the mineralization increased significantly. Therefore, we found that the dentine of the mandibular first molar of the rabbits was divided into three categories, i.e., AEFC, CIFC, and CMSC, by observing the rabbit molar teeth at 50 days and 8 months of age.

### 3.6 Cell proliferation and apoptosis

**FIGURE 6:**
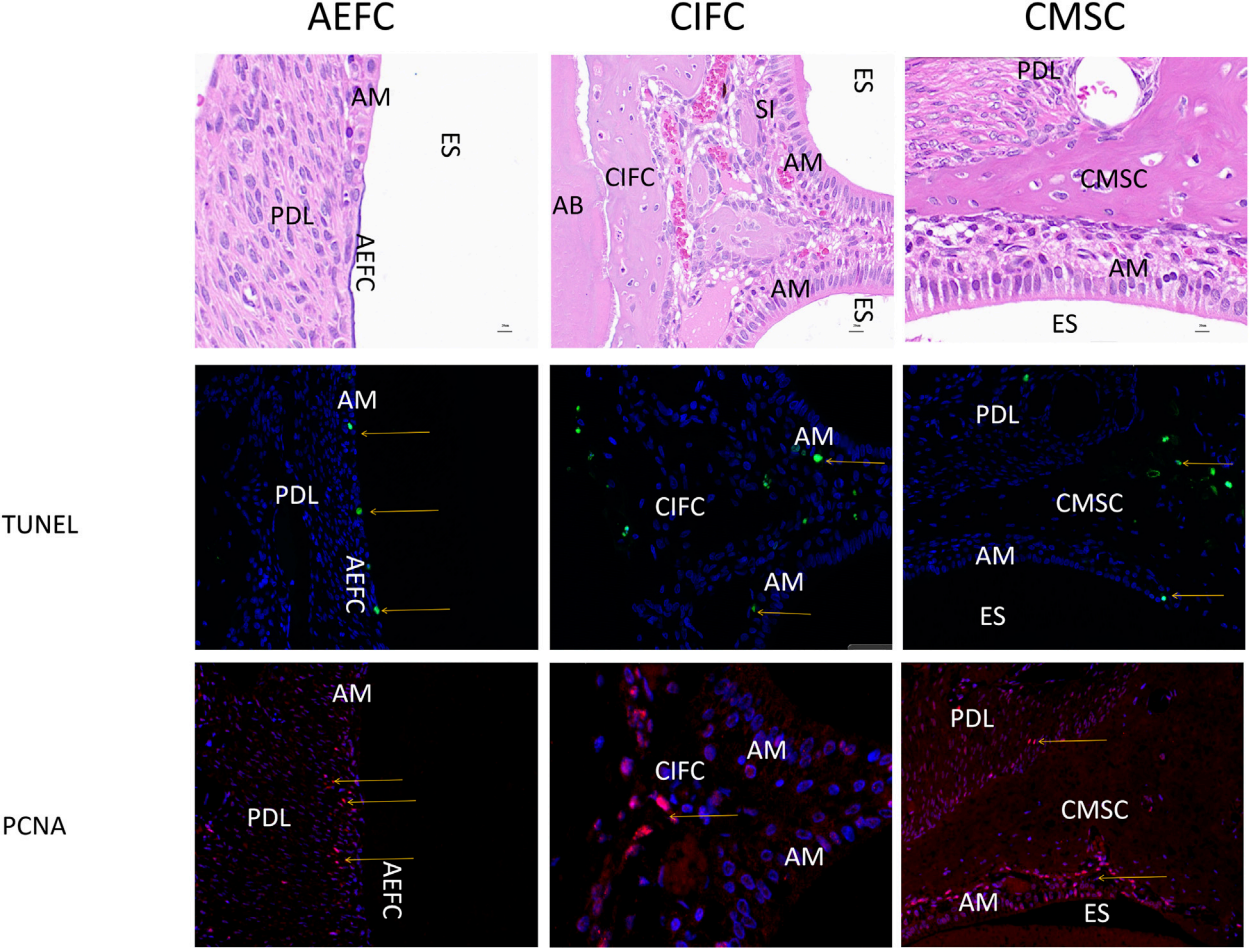
Expression images of PCNA and TUNEL in 50-day rabbit mandibular first molars Note: Yellow arrows indicate positive expression in both epithelial and mesenchymal cells. AM: Ameloblasts; AEFC: Acellular extrinsic fiber cementum; AB: Alveolar bone; CIFC: Cellular intrinsic fiber cementum; CMSC: Cellular mixed stratified cementum; D: Dentin; ES: Enamel space; PDL: Periodontal ligament; scale bar = 20 μm.

TUNEL and PCNA were examined for their expression in the above three locations of the mandibular first molar of 50-day rabbits, TUNEL and PCNA were stained green and red, respectively. TUNEL signals of the three locations as shown by the yellow arrows were all found in the inner part of the epithelial cells, so that apoptosis may exist in the epithelial cells (Figure 6). While no obvious TUNEL-positive cells were seen in the odontoblasts, the expression of PCNA in the three locations as shown by the red arrows, PCNA-positive cells showed a large number of expression signals at the junction line between the epithelial cells and mineralized odontoblasts, which suggests that the neoplastic region of our mineralized odontoblasts is located at the junction of the epithelial cells and the odontoblasts.

### 3.7 Expression of molecular markers

**FIGURE 7:**
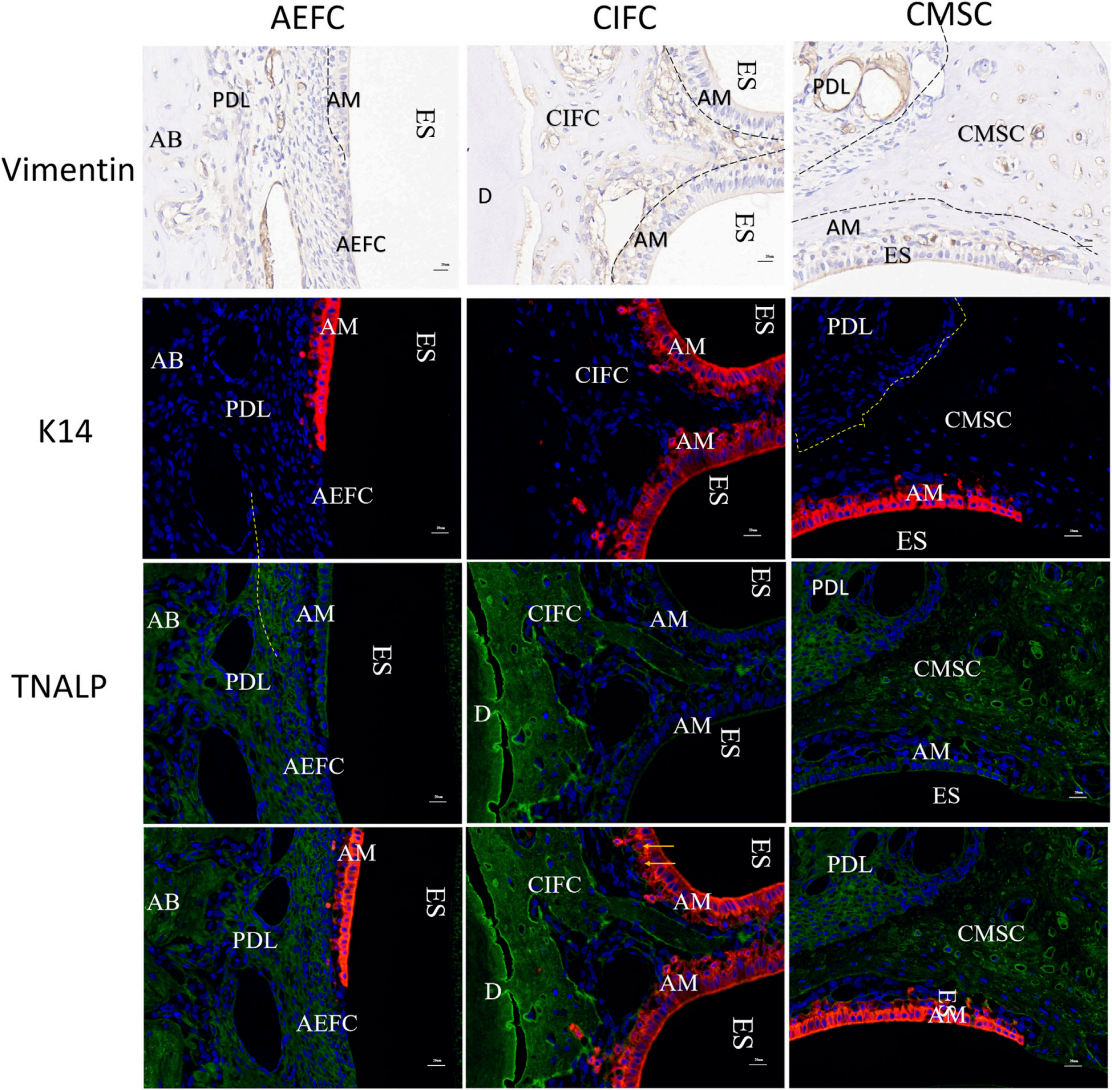
Vinmentin, K14, TNALP, K14-TNALP staining Note: AM: Ameloblasts; AEFC: Acellular extrinsic fiber cementum; AB: Alveolar bone; CIFC: Cellular intrinsic fiber cementum; CMSC: Cellular mixed stratified cementum; D: Dentin; ES: Enamel space; PDL: Periodontal ligament; scale bar = 20 μm.

Vimentin is not expressed in the outer acellular extrinsic fiber cementum (AEFC) or in the cellular mixed stratified cementum (CMSC) pulled by periodontal ligament fibers; however, in the embedded cellular intrinsic fiber cementum (CIFC) area and the outer CMSC, there are cells with vimentin-positive staining in the undifferentiated epithelial cell layer. Cytokeratin 14 (K14) was positively expressed in epithelial cells, Tissue-nonspecific alkaline phosphatase (TNALP) was expressed in the periodontal ligament and cementoblasts; however, K14-positive epithelial cells exhibited minimal immunoreactivity to TNALP. Double immunofluorescence staining revealed TNALP expression in non-apoptotic Hertwig’s epithelial root sheath (HERS) cells and in vascular-like structures within the CIFC region. Meanwhile, we did not observe yellow cells with dual expression of K14 and TNALP in the results of double staining (Figure 7).

## 4 Discussion

### 4.1 Morphogenesis of cementum

The primary function of cementum is to provide anchorage for the fibers of the PDL, thereby securing the tooth within the alveolar bone. In mammals, teeth are suspended in the alveolar socket via cementum and associated periodontal tissues, which facilitates tooth attachment, prolongs tooth longevity, and maintains proper occlusal relationships. Based on paleontological studies of these tissues and the presence of associated Sharpey’s fibers in many extinct and extant reptiles (including lizards), there is increasing support for the hypothesis that cementum, alveolar bone, and the periodontal ligament were originally present in all amniotes (including both mammals and reptiles) (LeBlanc et al., 2016). The presence of cementum is therefore considered characteristic of the thecodont dentition. In certain herbivorous mammals, the coronal portion may be covered by coronal cementum, which exists in two forms: one type fills the spaces between tall, flattened cusps and fuses them together, while the other type serves the same function as radicular cementum—covering the dentin of the root region where enamel is absent—by anchoring the periodontal ligament to the tooth and securing it within the alveolar bone (Boyde, 2005). Moriyama K (Ignaszak-Dziech et al., 2023) used scanning electron microscopy to examine the three-dimensional distribution and structural characteristics of three different types of cementum in the molars of guinea pigs, a species with hypselodont dentition. The molars of guinea pigs consist of two longitudinal, deeply infolded grooves. Except for the buccal surface of the upper molars and the lingual surface of the lower molars, all other surfaces are covered with enamel. In enamel-free regions, a continuous, band-like layer of acellular cementum was observed on the dentin surface. On enamel-covered surfaces, two distinct types of coronal cementum were identified: one type is distributed at nearly regular intervals around the peripheral enamel surface, from the apex to the occlusal surface; the other type is a cartilage-like cementum that nearly fills the entire occlusal portions of the two longitudinal grooves. These types of cementum may serve various functional roles, including mastication, anchorage, and continuous tooth eruption. Thus, the types of cementum vary considerably among different species.

In our experiment, various types of cementum are clearly present on the outer side of the enamel in rabbits. Micro-CT analysis revealed a progressive increase in the density and bone volume fraction (BV/TV) of cementum in rabbits as they aged. Through morphological and developmental investigations of the mandibular first molar cementum in rabbits, we identified three types of cementum: AEFC, CIFC, and CMSC. Comparative SEM observations of cementum distribution in mandibular first molars of 50-day-old and 8-month-old rabbits showed that the cementum of the mandibular first molars also undergoes growth and morphological changes over time, becoming increasingly denser and more voluminous. Masson staining performed on 50-day and 8-month-old rabbit specimens revealed the extent of mineralization and width increment of CMSC with PDL fiber insertions. Based on these findings, we hypothesize that only CMSC with fiber insertions can perform the functional role of cementum. The development of mandibular first molar cementum in rabbits is not constrained by the root morphology or the shape of enamel. Cementum is present in hypselodont teeth, regardless of root formation. The intermediate CIFC embeds within the enamel folding region, functioning as a filler. In animals with hypselodont and ever-growing teeth, the peripheral cementum serves to anchor the tooth within the alveolar bone, a function invariably associated with the insertion of PDL fibers. The growth of this peripheral cementum is related to the tensile force exerted by the PDL fibers. In contrast, the growth of internal cementum is independent of PDL fiber insertion and contributes to structural stabilization through gradual mineralization.

### 4.2 Cellular origins of cementum development

Root formation begins after the formation of the tooth crown, dentin, and enamel, and occurs prior to tooth eruption. The cervical loop represents the apical extension of the enamel organ and gives rise to the bilayered Hertwig’s epithelial root sheath (HERS), which is composed of the outer enamel epithelium (OEE) and inner enamel epithelium (IEE) (Suchetha et al., 2017). Cementogenesis initiates when HERS epithelial cells and mesenchymal cells from the dental follicle approach the developing root surface. Over the past few decades, extensive research has provided valuable insights into the roles of various tissues during root development; however, the respective contributions of epithelial and mesenchymal cells in cementum formation remain a subject of ongoing debate (Diekwisch, 2004). For a long time, the classical mesenchymal hypothesis—widely accepted in the field—has posited that cells from the dental follicle migrate to the developing root surface and differentiate into cementoblasts (Cho and Garant, 2000; Yamamoto et al., 2006; Yamamoto and Takahashi, 2009; Yamamoto et al., 2014; Yamamoto et al., 2015; Xie et al., 2019). This hypothesis aligns with the overarching concept that all three principal periodontal cell lineages originate from a common source: the dental follicle (Guo et al., 2012). However, during the 1980s and 1990s, an alternative hypothesis emerged based on *in vitro* and *in vivo* experiments conducted in rats and mice. According to this epithelial hypothesis, some cells from the epithelial root sheath are capable of undergoing epithelial-mesenchymal transition (EMT) and differentiating into cementoblasts (Foster et al., 2007; Foster, 2017). Thomas was the first to propose the epithelial hypothesis in an *in vitro* study, where he observed that cultured epithelial root sheath cells underwent morphological changes into a mesenchymal-like phenotype, co-expressing both vimentin and keratin—markers for mesenchymal and epithelial cells, respectively (Fitchett and Hay, 1989; Gibbins et al., 2002; Bosshardt and Schroeder, 1996) Based on these findings, he suggested that cultured HERS cells could undergo EMT to differentiate into cementoblast-like mesenchymal cells. Furthermore, during *in vivo* cementogenesis, HERS cells are also believed to transform into cementoblasts through EMT. Subsequently, numerous *in vivo* and *in vitro* experiments have supported the epithelial hypothesis (Lézot et al., 2000; Sonoyama et al., 2007; Huang et al., 2009) However, in vivo studies, cementoblasts and epithelial cells may have been misidentified. This is because certain cementoblasts and cementocytes exhibit immunoreactivity for keratin, an epithelial marker, leading to the interpretation that these were HERS cells actively undergoing EMT at the time. Some studies have confirmed that the epithelial cell rests of Malassez coexist with true cementoblasts on the cementum surface, and that numerous epithelial cells become embedded within the cellular cementum (Alsulaimani, 2016). Consequently, the epithelial rests of Malassez and embedded epithelial cells might have been misidentified as cementoblasts and cementocytes, respectively. The various fates of HERS following its fragmentation have been demonstrated: incorporation as epithelial rests of Malassez (ERM) contributing to the maintenance of the PDL (Rosset et al., 2017; Silva et al., 2017; Koidou et al., 2018), cellular death through apoptosis (Chen et al., 2014), or participation in the formation of cementum and the periodontal ligament either as mesenchymal cells via EMT (Gibbins et al., 2002) or as epithelial cells (Yang et al., 2014; Zakrzewski et al., 2019). Although substantial progress has been made in elucidating the origin of cementum and numerous divergent viewpoints have been presented in the literature, the origin of cementum remains a subject of debate.

In our experiments, three types of cementum were observed in the mandibular first molars of rabbits. Among them, the appearance of AEFC occurred after epithelial cell apoptosis, and its appositional growth is likely primarily associated with the proliferation of cementoblasts located in the periodontal ligament near the cementum surface and the traction of collagen fibers. Analysis of PCNA and TUNEL results in CMSC and CIFC suggests that apoptosis occurs within the epithelial cell layer of the rabbit mandibular first molars, while cellular proliferation is concentrated at the interface between the epithelial cells and mineralized cementum. Although epithelial cells and mineralized cementum coexist in this region, the mineralized cementum and mature ameloblasts are still separated by undifferentiated epithelial stem cells and mesenchymal stem cells. Therefore, we speculate that, after a certain period of growth, epithelial cells in the mandibular first molars of rabbits gradually undergo degeneration and apoptosis. Meanwhile, the layer of undifferentiated proliferative cells exhibits active growth, progressively generating newly formed mineralized cementum that replaces the apoptotic epithelial cells. This conclusion is consistent with the observations by Yamamoto T (Yamamoto et al., 2014) in the developmental study of cementum in rats. Accordingly, we propose that epithelial cells are not the direct cellular source of cementum. However, apoptotic epithelial cells or undifferentiated epithelial stem cells are indeed present around newly formed cementum. It is thus inferred that signals released during the apoptosis of epithelial cells or epithelial stem cells may stimulate the differentiation of mesenchymal stem cells toward a cementoblastic lineage, thereby contributing to the formation of mineralized cementum.

### 4.3 Does EMT occur during cementum development?

The epithelial hypothesis—which posits that the formation and development of cementum involve EMT of the HERS—has been refuted in numerous *in vivo* studies. For instance, Yamamoto T (Yamamoto and Takahashi, 2009), aiming to clarify whether EMT occurs in HERS, utilized a double immunohistochemical approach involving cytokeratin–vimentin and runt-related transcription factor 2 (Runx2)–cytokeratin staining to examine developing molars in rats. During both acellular and cellular cementum formation, HERS and its epithelial derivatives expressed cytokeratin but did not express vimentin or Runx2. In contrast, vimentin and Runx2 were expressed in dental follicle cells and cementoblasts, while cytokeratin was not expressed in these cells. No cells co-expressing cytokeratin–vimentin or Runx2–cytokeratin were detected. These findings indicate the absence of an intermediate phenotype indicative of epithelial-to-mesenchymal transformation in epithelial cells. Moreover, HERS cells do not contribute to the production of mineralized tissues. That is, during the formation of both acellular and cellular cementum in rats, the HERS does not undergo epithelial-mesenchymal transition. Yamamoto T (Yamamoto et al., 2006) investigated the development of acellular cementum in rat molars using immunohistochemical methods, employing antibodies against three matrix metalloproteinases that degrade epithelial root sheath maintenance factors (i.e., basement membrane and desmosomes) to study the proteolytic activity of the HERS. Tissue-nonspecific alkaline phosphatase (TNALP) and keratin were used to detect EMT. HERS cells exhibited immunoreactivity to all three enzymes during fragmentation, suggesting that the disintegration of HERS is enzyme-mediated. Dental follicle cells and cementoblasts showed strong immunoreactivity to TNALP, with the reaction extending from the alveolar bone-associated region to the root-associated region. There were very few cells that exhibited double immunoreactivity for keratin and TNALP. Keratin-positive HERS cells showed negligible TNALP immunoreactivity, and epithelial cells appeared not to migrate into the dental follicle. These findings suggest the absence of a transitional phenotype between epithelial cells and cementoblasts in the developing dental follicle, and therefore indicate that HERS cells do not undergo EMT during the initial formation of acellular cementum. Similarly, in our study, results from double staining with vimentin and K14-TNALP did not yield definitive evidence supporting the EMT hypothesis. In the present study, no vimentin expression was detected on the outer surfaces of both the AEFC and the CMSC; however, positive expression was observed in undifferentiated cells within the region of CIFC, which may suggest that undifferentiated epithelial cells produced vimentin through an EMT process. In the double-staining results of K14 and TNALP, no positive expression was observed on the outer surfaces of AEFC and CMSC adjacent to the PDL, indicating that the growth of these cementum types is unrelated to EMT. In contrast, in the CIFC region, TNALP-positive signals were present along the vascular endothelium and among the proliferating epithelial cells. However, no overlapping signals of K14 and TNALP were observed in any single epithelial cell. These results provide no definitive evidence for the occurrence of EMT during cementum development.

Interestingly, in rabbit molars, the cementum in contact with the PDL appears to develop independently of epithelial cells. The growth and mineralization of CIFC, which is closely associated with epithelial cells, do not contribute to the attachment of the tooth to the PDL or to the fixation of the tooth within the alveolar bone. Therefore, even if EMT does occur and epithelial cells are transformed into cementoblasts, this portion of cementum, lacking insertion of extrinsic fibers, would still be unable to fulfill the functional role of the periodontal complex.

## Data Availability

The raw data supporting the conclusions of this article will be made available by the authors, without undue reservation.
